# Valve Expectoration After Endobronchial Valve Placement: Potential Utility of Computed Tomography‐Based Airway Sizing—A Case Report

**DOI:** 10.1002/rcr2.70817

**Published:** 2026-11-09

**Authors:** Daisuke Kinose, Yukihiro Nagatani, Hiroaki Nakagawa, Mayu Fujino, Amane Ichida, Reo Tanaka, Shogo Okuda, Yasumitsu Ueki, Masashi Misonou, Akio Yamazaki, Emiko Ogawa, Masafumi Yamaguchi, Yasutaka Nakano

**Affiliations:** ^1^ Department of Respiratory Medicine Shiga University of Medical Science Otsu Shiga Japan; ^2^ Department of Radiology Shiga University of Medical Science Otsu Shiga Japan; ^3^ Health Administration Center Shiga University of Medical Science Otsu Shiga Japan

**Keywords:** airway calibre, chronic obstructive pulmonary disease, CT‐based airway sizing, emphysema, endobronchial valve expectoration

## Abstract

A 75‐year‐old man with chronic obstructive pulmonary disease underwent endobronchial valve (EBV) placement in the left lower lobe and expectorated the valve from left B10 1 month later. Retrospective analysis of preprocedural dynamic computed tomography (CT) images showed that the median B10 luminal diameter increased from 4.25 mm at end‐expiration to 6.55 mm at end‐inspiration, approaching the upper limit of the recommended working range (4.0–7.0 mm) for the initially placed size 4.0 valve. A size 5.5 valve was subsequently placed without further expectoration. Bronchoscopic sizing under general anaesthesia with neuromuscular blockade and positive‐pressure ventilation may underestimate the maximum airway calibre encountered during spontaneous breathing, particularly in dependent bronchi. Inspiratory CT‐based airway assessment may provide useful complementary information for planning EBV placement and selecting an appropriate valve size.

## Graphical abstract

After a 75‐year‐old man with COPD expectorated an endobronchial valve, retrospective analysis of preprocedural dynamic CT showed left B10 diameter increasing from 4.25 mm at end‐expiration to 6.55 mm at end‐inspiration, near the size 4.0 valve's upper working limit. Inspiratory CT may aid valve placement planning and size selection.

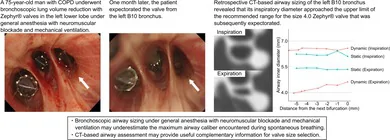

## 1 Introduction

Endobronchial valves (EBVs) are used for bronchoscopic lung volume reduction in selected patients with severe emphysema. Valve migration and expectoration are uncommon but clinically relevant complications [1]. We report a case of EBV expectoration in which retrospective computed tomography (CT) measurements revealed respiratory variation in airway calibre, suggesting that bronchoscopic sizing under general anaesthesia with neuromuscular blockade and positive‐pressure ventilation may not reflect airway calibre during spontaneous breathing.

## 2 Case Report

A 75‐year‐old man with chronic obstructive pulmonary disease (COPD) and a 60‐pack‐year smoking history was referred for EBV treatment because of persistent exertional dyspnoea despite dual long‐acting bronchodilator therapy and pulmonary rehabilitation. His modified Medical Research Council (mMRC) dyspnoea grade was 3. Pulmonary function testing showed severe airflow obstruction with marked hyperinflation and reduced diffusing capacity (Table 1). CT demonstrated lower‐lobe‐predominant emphysema, and perfusion scintigraphy showed reduced perfusion of the left lower lung. The left lower lobe was therefore selected as the treatment target.

**TABLE 1:** Pulmonary function tests.

	Measurements (% predicted)
FVC	3.14 L (84.0%)
FEV_1_	1.00 L (33.5%)
FEV_1_/FVC	32.0%
DL_CO_	5.16 mL/min/mmHg (39.4%)
DL_CO_/V_A_	0.91 mL/min/mmHg/L (21.5%)
TLC	12.4 L (190.9%)
RV	9.50 L (355.5%)
RV/TLC	76.6%

Abbreviations: DL_CO_, diffusing capacity of the lung for carbon monoxide; FEV_1_, forced expiratory volume in 1 s; FVC, forced vital capacity; RV, residual volume; TLC, total lung capacity; V_A_, alveolar volume.

Under general anaesthesia with neuromuscular blockade and mechanical ventilation, the absence of collateral ventilation between the left upper and lower lobes was confirmed. Bronchoscopic sizing with the dedicated catheter led to placement of size 4.0 Zephyr regular valves (Pulmonx Corporation, Redwood City, CA, USA) in left B8, B9 and B10 (Figure 1C), and a size 5.5 low‐profile valve in left B6. Dyspnoea improved to mMRC Grade 1, although complete left lower‐lobe atelectasis was not achieved. One month later, the patient expectorated an EBV following a coughing episode, and his dyspnoea returned to Grade 3. CT and bronchoscopy confirmed loss of the valve from left B10 (Figure 1B,D). A size 5.5 regular valve was subsequently placed in B10. No further valve migration or expectoration has occurred, although complete left lower lobe atelectasis was not achieved.

**FIGURE 1:**
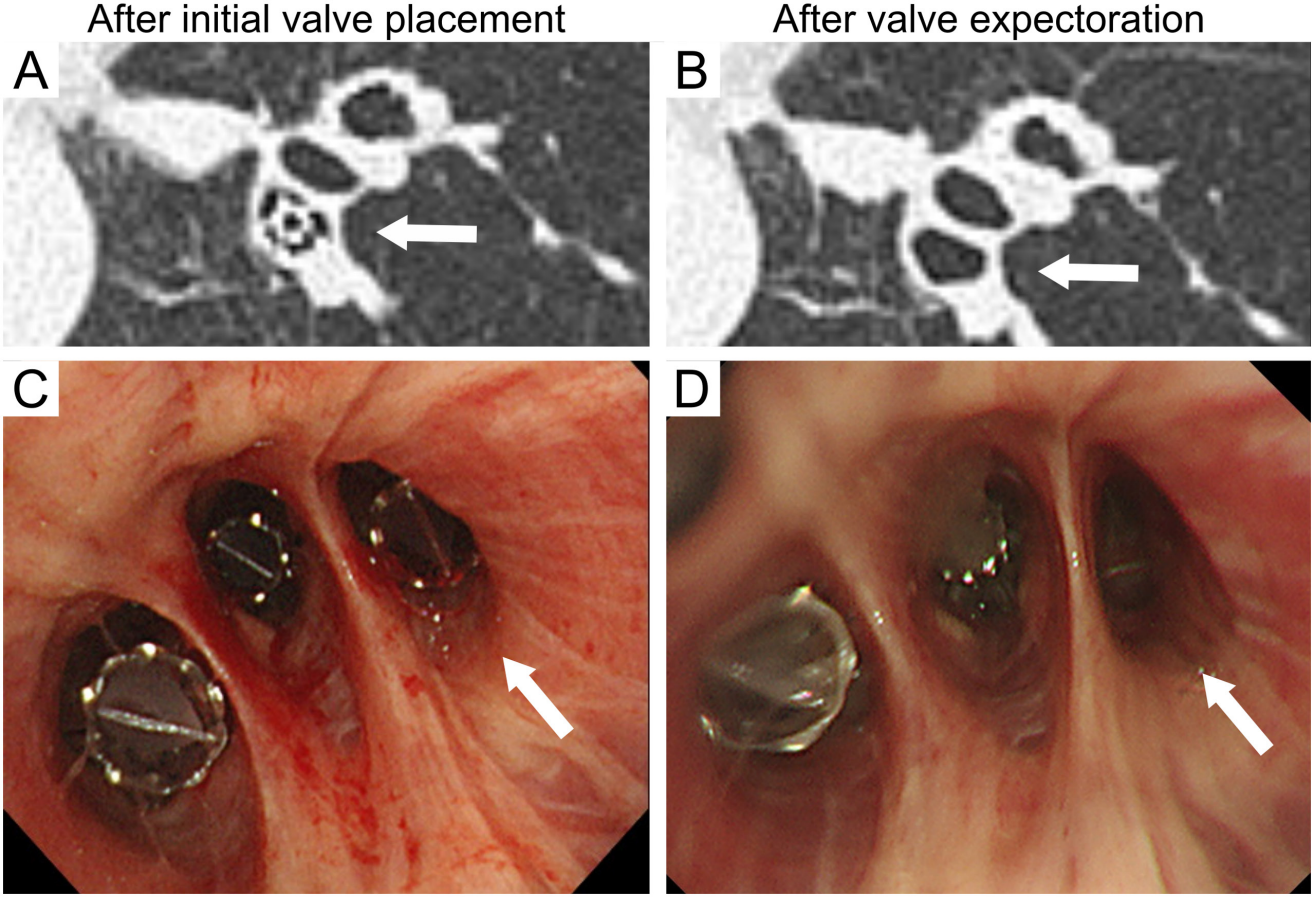
CT and bronchoscopic findings after initial valve placement and after expectoration of the left B10 valve. CT images of the left B10 (A, B) bronchus and bronchoscopic views of the left lower‐lobe basal bronchi (C, D). The left panels show findings after initial valve placement and the right panels show findings after valve expectoration. Arrows indicate the left B10 bronchus.

Valve undersizing, variation in airway calibre, inadequate landing‐zone length, suboptimal placement and granulation tissue have been associated with EBV migration [1]. As the B10 bronchus had an adequate landing zone and no granulation tissue was observed after expectoration, we investigated whether airway–valve size mismatch had contributed to valve displacement. Preprocedural inspiratory, expiratory and dynamic ventilation CT images were therefore retrospectively analysed to quantify airway lumen dimensions. Cross‐sectional images perpendicular to the bronchial axis were reconstructed along the distal approximately 5 mm of each target bronchus. The airway luminal boundary was determined using the full‐width at half‐maximum method [2] and a perimeter‐derived inner diameter was calculated as luminal perimeter divided by π.

On static CT, the median [range] B10 diameter was 6.20 [6.11–6.42] mm during inspiration and 5.38 [5.33–5.43] mm during expiration (Figure 2C). Dynamic ventilation CT demonstrated substantially greater respiratory variation (Figure 2A,B), with the median B10 diameter changing from 6.55 [6.45–6.59] mm at end inspiration to 4.25 [3.96–4.62] mm at end expiration (Figure 2C). Thus, the B10 lumen approached the lower end of the size 4.0 valve range during expiration but expanded close to the upper end of its recommended 4.0–7.0‐mm working range during inspiration. The replacement size 5.5 valve has a recommended working range of 5.5–8.5 mm.

**FIGURE 2:**
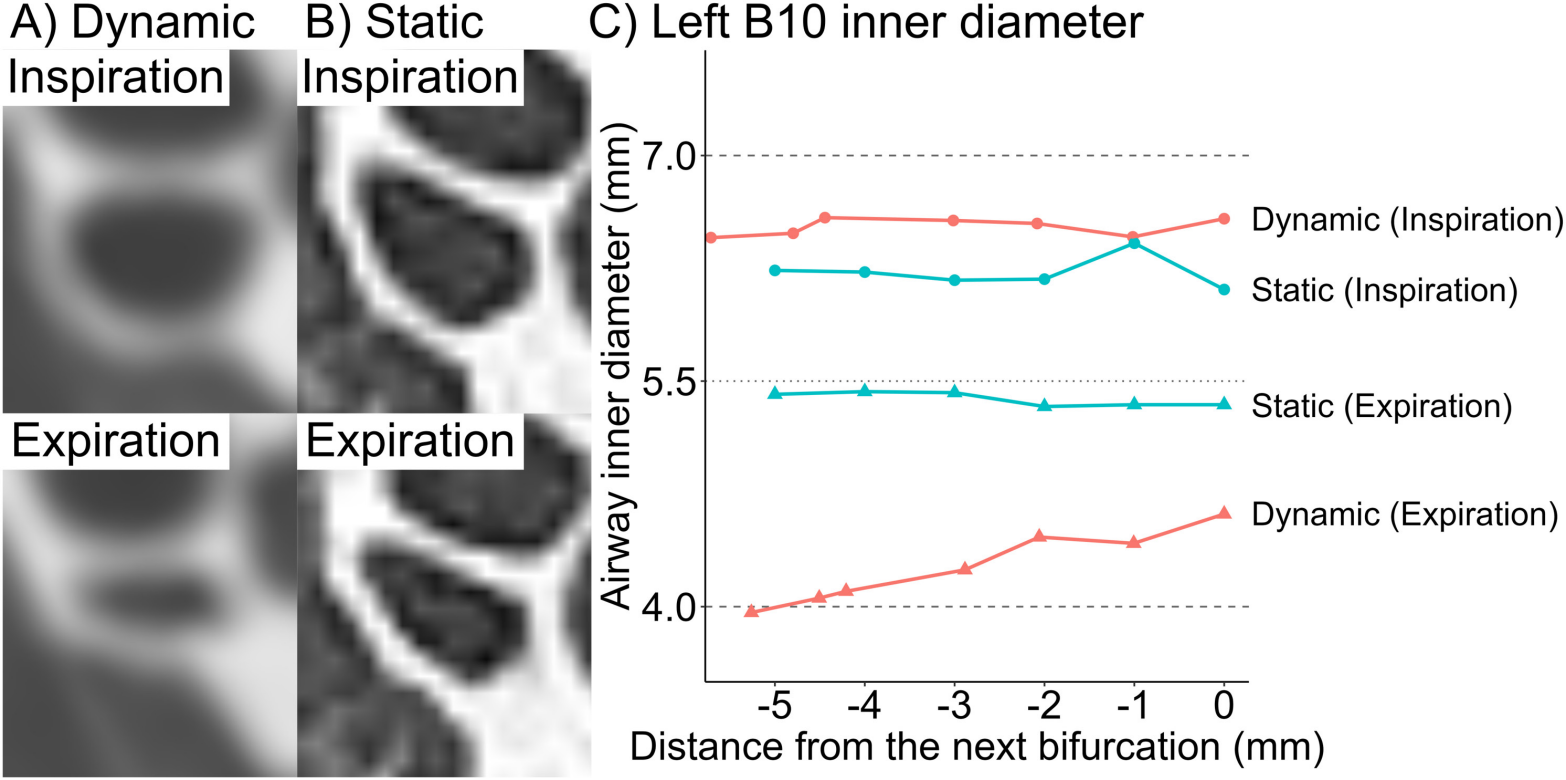
Respiratory changes in the calibre of the left B10 bronchus assessed by static and dynamic CT. (A) Cross‐sectional images of the left B10 bronchus at the end‐inspiratory and end‐expiratory phases of dynamic ventilation CT. (B) Cross‐sectional images of the left B10 bronchus on static inspiratory and expiratory CT. (C) Airway luminal diameters of the left B10 bronchus measured at the end‐inspiratory and end‐expiratory phases of dynamic ventilation CT and static inspiratory and expiratory CT. A perimeter‐derived inner diameter was calculated as the inner luminal perimeter divided by *π*. Red and blue lines indicate dynamic ventilation CT and static CT, respectively; circles and triangles indicate inspiration and expiration, respectively. Dashed lines indicate the lower and upper limits of the recommended working range for the size 4.0 Zephyr valve (4.0–7.0 mm) and the dotted line indicates the lower limit of the recommended working range for the size 5.5 Zephyr valve (5.5–8.5 mm).

## 3 Discussion

Airway calibre changes with lung volume because of changes in transmural pressure and parenchymal traction. Previous CT studies have demonstrated greater respiratory variation in lower‐lobe than upper‐lobe bronchi [3]. Gravitational dependence may further influence airway dynamics [4]. General anaesthesia with neuromuscular blockade reduces functional residual capacity and promotes dependent airway narrowing and atelectasis [5]. Positive‐pressure ventilation also alters regional ventilation. As left B10 is dependent in the supine position, bronchoscopic sizing under these conditions may have sampled a relatively small, expiratory‐like airway calibre. In contrast, routine preprocedural inspiratory CT is acquired while the patient is awake at a higher lung volume and may more closely approximate the maximum airway calibre encountered during spontaneous breathing than bronchoscopic sizing under general anaesthesia.

The inspiratory B10 diameter remained within the recommended working range for the size 4.0 valve. We therefore cannot establish undersizing as the cause of expectoration. Nevertheless, the combination of marked respiratory calibre variation, persistent distal aeration after the initial procedure and a coughing episode could have reduced valve apposition sufficiently to facilitate displacement. The absence of further valve migration or expectoration after replacement with a size 5.5 valve suggests that selecting a slightly larger valve may improve valve retention. In fact, in real‐world clinical practice, 60% of implanted valves were reported to be oversized relative to retrospectively measured CT‐derived airway diameters [6].

Importantly, inspiratory CT is already routinely obtained to assess emphysema distribution and fissure completeness before EBV treatment. Reviewing the target airway on these existing images would therefore add no radiation exposure and could provide complementary information for planning EBV placement and selecting an appropriate valve size, particularly when the airway diameter approaches a valve‐size boundary. Expiratory or dynamic CT may be useful when marked airway collapsibility is suspected but is unlikely to be necessary for routine sizing.

This observation has important limitations. It represents a single patient, the perimeter‐derived CT diameter has not been validated for EBV size selection and airway calibre was not measured simultaneously by CT and bronchoscopy under identical physiological conditions. The proposed effect of anaesthesia on B10 calibre therefore remains mechanistic inference rather than direct proof. Prospective comparison of bronchoscopic and CT‐based airway measurements is required.

In summary, this case illustrates that target bronchial calibre can vary substantially during respiration and that bronchoscopic sizing under general anaesthesia may not reflect the maximum airway calibre encountered during spontaneous breathing. Inspiratory CT‐based airway assessment should not replace bronchoscopic sizing, but may provide a useful complementary check when the measured airway lies near the upper limit of the recommended range for the selected EBV.

## Author Contributions

**Daisuke Kinose:** conceptualization, methodology, software, data curation, investigation, validation, formal analysis, visualization, project administration, writing – original draft, writing – review and editing. **Yukihiro Nagatani:** conceptualization, methodology, software, writing – review and editing. **Hiroaki Nakagawa:** writing – review and editing, data curation. **Mayu Fujino:** data curation, writing – review and editing. **Amane Ichida:** data curation, writing – review and editing. **Reo Tanaka:** data curation, writing – review and editing. **Shogo Okuda:** data curation, writing – review and editing. **Yasumitsu Ueki:** data curation, writing – review and editing. **Masashi Misonou:** data curation, writing – review and editing. **Akio Yamazaki:** data curation, writing – review and editing. **Emiko Ogawa:** writing – review and editing. **Masafumi Yamaguchi:** writing – review and editing. **Yasutaka Nakano:** methodology, software, formal analysis, visualization, writing – review and editing, investigation, supervision, resources.

## Funding

The authors have nothing to report.

## Consent

The authors declare that written informed consent was obtained for the publication of this manuscript and accompanying images using the form provided by the Journal.

## Conflicts of Interest

Daisuke Kinose, Reo Tanaka, Masashi Misonou, Yasumitsu Ueki and Akio Yamazaki report honoraria from AstraZeneca. Hiroaki Nakagawa reports honoraria from AstraZeneca and Boehringer Ingelheim. Masafumi Yamaguchi reports honoraria from AstraZeneca, GSK and Sanofi and fees for participation on an advisory board for Boehringer Ingelheim. Yasutaka Nakano reports honoraria from Sanofi, AstraZeneca, Pulmonx Corporation, Boehringer Ingelheim and GlaxoSmithKline. The other authors declare no conflicts of interest. The Department of Respiratory Medicine, Shiga University of Medical Science, has received institutional payments from Boehringer Ingelheim for clinical trial activities, from Pulmonx Corporation and GlaxoSmithKline for post‐marketing surveillance, from AstraZeneca for adverse‐event reporting and an institutional donation from Pulmonx Corporation. All of these relationships were outside the submitted work.

## Data Availability

The data supporting the findings of this case report are not publicly available and cannot be shared due to patient privacy and ethical restrictions.
